# Intelligence

**Published:** 1810-07

**Authors:** 


### *INTELLIGENCE.*

The Editors of the Medical and Physical Journal have the pleasure to inform their Readers, that the "Bill for the improvement of the Medical and Surgical and Veterinary Sciences, and for regulating the practice thereof," will be introduced into Parliament at the commencement of next Session, with every prospect of success. The Editors have further to observe, that although the several important objects of the Plan will be accomplished, they have good reason to believe, that no tax of any kind is intended to be imposed upon the present race of medical men. It was originally proposed to build the School of Physic, and otherwise improve Medical Science, by calling upon the Members of the Profession, who are not of the Corporate Bodies, for an equivalent, in in lieu of the admission fine; and this would, it must be admitted have been an equitable assessment. However, through the indefatigable exertions of the chief promoter of the scheme, and the liberality of government, it is now confidently expected, that all the objects of the Bill will be obtained on principles the most liberal towards the Profession at large. This subject is to be resumed in a future Number, when the further proceedings shall be communicated more in detail.

There have been vaccinated at the eight different stations of the National Vaccine Establishment in the Metropolis, 1493 persons; and the Directors have very lately found it expedient to establish a ninth station in the populous parish of Spital-Fields. The demand for the charges of vaccine fluid has been progressively increasing for several months, and since the last statement, the number of them delivered amounts to 16,749; which affords a strong presumption, that the practice of Vaccination is becoming more and more extended; an inference abundantly confirmed by the accounts which have been transmitted to the Board from all parts of the empire.

There is now a boy, eight years old, in Old Gravel Lane, who is recovering from small-pox, which he has had pretty severely *a second time.* The first time was about three years ago, when he caught them, together with his sister, from a child who was inoculated in the house where they dwelt. The boy and girl both sickened together, both had a plentiful eruption, were both blind during the disease, and were marked by the pustules. From what source the boy has now taken them cannot be ascertained, but the pustules are very numerous, and have gone through complete maturation, as on the former occasion. The girl remains uninfected.

Mr. de Luc has invented a machine which he denominates the Electric Column, and which, by some of our natural philosophers, is considered the most important discovery in the science of electricity, since that of the Voltaic pile. He is preparing an account of it for publication.

An improved method of preparing phosphorus bottles. The phosphorus being carefully dried by filtering paper, cut a thin slice, divide it into as many pieces as can expeditiously be done, and introduce each piece into a small bottle, with as much lime as will surround it. Lime slaked in the air and submitted to a strong red heat, in a black lead crucible, for twenty minutes, is in a good state for the purpose. The bottle, when full, may be exposed, corked, to the radiant heat of a fire, till some of the pieces of phosphorus have assumed an orange tint. It will then be ready for immediate use. But the heating is not necessary, if the bottle is not wanted for immediate use, and it will continue longer in a serviceable state. In using the bottle, the mouth should be closed as the match is withdrawn. Bottles thus prepared continue serviceable four or five months, though very frequently used.

Mr.

Mr. Knight, in his report of the transactions of the Horticultural Society, mentions an improved method of cultivating the alpine strawberry. The process consists of sowing the seed on a moderate hot-bed, in the beginning of April, and removing the plants as soon as they have acquired sufficient strength, to beds in the open ground. They will begin to blossom after Midsummer, and afford an abundant late autumnal crop. Mr. K. thinks, that this strawberry ought always to be treated as an annual plant.

The following curious circumstance respecting the toad, is communicated by a correspondent to Nicholson's Journal:—"A person," says he, "in the neighbourhood of Maidstone, who manufactures brown paper, informed me, while I was observing his people at work, that he had frequently placed a toad amidst a pile of sheets to be pressed, and always found it alive and well on taking out, though it must have sustained with the paper a pressure equivalent to several tons; but a frog could never survive the same degree of pressure. I sought a long time for a toad to see the experiment myself, but was unable to find one till after the men had left work."

The cranium of a horrid animal, the race of which seems to be extinct, has been recently dug up near Minava. From the description given of this part of the skeleton, the animal must have been at least ten or twelve feet long. The horns which are attached to the head, and have partly passed into a fossil state, far exceed in size those of the oxen of the present day. They are a foot and a half in circumference at the root, and two feet and a half long. It was hoped that the entire skeleton would be recovered; but on further search, two teeth only were found. Foreign naturalists are of opinion, that this head must have belonged to the race of Urus or Aurochs, mentioned by Cæsar in his Commentaries, and which some even suppose still to exist in the mountains of Siberia, and in the forests of Poland.

In addition to the circumstances already detailed respecting the late earthquakes at the Cape of Good Hope, the following particulars are communicated in a letter, dated Cape Town, January 1810. My last letter was principally about earthquakes, which have been repeated almost every day since the 4th ult. During the last week we have had five or six shocks, but none except the three on December 4, and two since, have been violent. The Dutch inhabitants begin to console themselves with the idea that the noises we hear are thunder, although not a cloud is to be seen in any part of the sky. These earthquakes have greatly reduced the value of houses, most of which in the colony are more or less damaged. In every part of the settlement the shocks have been experienced, in some slightly, in others in a more violent degree. Salt water has been thrown up in places at the distance of three or four miles from the sea, without leaving any appearance of springs or openings in the soil. In other parts, where the soil is black, as low down as our wells have been dug, several spots of white sand, about about six feet in diameter, and generally of a circular form, have been thrown up, evidently in union with water, which immediately subsided. Springs of water have also burst out in many parts of the colony where there never were any before. A waggon, which came into Cape Town two days ago, sunk to the top of the wheels in a quick-sand, which is thrown up in the middle of a road that was before as hard as a rock. If these are the only effects that will be produced by such subterraneous convulsions, we have great reason to be satisfied with the result, since our climate appears to have been greatly ameliorated by them. Ever since the first shocks, we have experienced cool pleasant weather, and have been free from those violent winds, which at this season of the year, usually prevailed three days out of seven. During the last month, which is our Midsummer, the thermometer has seldom been higher than 72°, and the barometer has varied between 29·30 and 30·15. Our winter passed with only one storm of thunder and lightening, and that by no means violent. The first winter of my arrival (1808), I believe we had thunder two or three times a week, for five weeks successively. If, as some philosophers assert, electricity be the cause of earthquakes, may it not also account for the absence of thunder and lightening, which we have experienced during the last winter?

---

Dr. Clough, Physician Man-midwife to the St. Mary-le-bone General Dispensary, &c. will, on Monday the 6th of August, at ten in the morning, commence his next Course of Lectures on the Science and Practice of Midwifery, including the Diseases of Women and Infants.

Dr. Squire will begin a Course of Lectures on Midwifery and the Diseases of Women and Children, on Tuesday, July 3, 1810. For particulars apply to Dr. Squire, 30, Ely Place, Holborn.

